# A systematic review and meta-analysis of structural magnetic resonance imaging studies investigating cognitive and social activity levels in older adults

**DOI:** 10.1016/j.neubiorev.2018.06.012

**Published:** 2018-10

**Authors:** M. Anatürk, N. Demnitz, K.P. Ebmeier, C.E. Sexton

**Affiliations:** a Department of Psychiatry, University of Oxford, Warneford Hospital, Warneford Lane, Oxford, OX3 7JX, United Kingdom; b Wellcome Centre for Integrative Neuroimaging, Oxford Centre for Human Brain Activity, Department of Psychaitry, University of Oxford, Warneford Hospital, Warneford Lane, Oxford, OX3 7JX, United Kingdom; c Global Brain Health Institute, Memory and Aging Center, Department of Neurology, University of California, San Francisco, CA, 94158, USA

**Keywords:** Brain, Social activity, Cognitive activity, Aging, Magnetic resonance imaging, Voxel-based morphometry, Region-of-interest, Gray matter, White matter

## Abstract

Population aging has prompted considerable interest in identifying modifiable factors that may help protect the brain and its functions. Collectively, epidemiological studies show that leisure activities with high mental and social demands are linked with better cognition in old age. The extent to which socio-intellectual activities relate to the brain’s structure is, however, not yet fully understood. This systematic review and meta-analysis summarizes magnetic resonance imaging studies that have investigated whether cognitive and social activities correlate with measures of gray and white matter volume, white matter microstructure and white matter lesions. Across eighteen included studies (total n = 8429), activity levels were associated with whole-brain white matter volume, white matter lesions and regional gray matter volume, although effect sizes were small. No associations were found for global gray matter volume and the evidence concerning white matter microstructure was inconclusive. While the causality of the reviewed associations needs to be established, our findings implicate socio-intellectual activity levels as promising targets for interventions aimed at promoting healthy brain aging.

## Highlights

• Socio-intellectual activity correlates with global white matter volume and lesions.
• Volumetric measures of the hippocampus are linked with non-physical activities.
• Local gray matter volume in each cerebral lobe also relates to leisure activities.

## 1 Introduction

The proportion of older adults is rapidly growing worldwide, with concurrent increases in the number of individuals living with Alzheimer’s disease (Prince et al., 2015). As a result, there is considerable interest among the scientific community to identify modifiable factors that promote healthy cognitive aging, with the overarching aim to develop interventions that slow, delay or prevent cognitive impairment in old age. These research efforts have been fruitful, with activities undertaken during an individual’s leisure time emerging as one of several potentially protective factors (Fratiglioni et al., 2004; Kuiper et al., 2016; Wang et al., 2012; Yates et al., 2016). Benefits have been reported for both cognitive activities (CA), which predominantly require mental engagement with minimal interpersonal interactions, and social activities (SA), where the core feature is socializing with others (Seider et al., 2016). For example, a meta-analysis conducted by Yates et al. (2016) identified CA as significantly correlated with a lower risk of cognitive decline and dementia, higher levels of global cognition and better performance on tests of executive functioning, memory, processing speed and language. Infrequent participation in SA has also been related to more rapid mental decline (Kuiper et al., 2016). While the link between leisure activities and old age cognition is well established, the neurobiological mechanisms supporting these associations are not yet fully understood.

Neuroimaging techniques provide a powerful tool for examining the neural mechanisms underlying the link between leisure activities and old age cognition (Bartrés-Faz and Arenaza-Urquijo, 2011). Neuroimaging studies published within this area broadly fall into three categories: (1) structural magnetic resonance imaging (MRI), (2) functional MRI (fMRI) and (3) Positron emission tomography (PET)/Single-photon emission computed tomography (SPECT) imaging. Structural MRI measures the neuroanatomical properties of the brain, including global and regional gray matter (GM) and white matter (WM) volume, WM microstructure and WM lesions (Giorgio et al., 2010). These structural features have been proposed as indices of *‘brain reserve’* (Stern, 2002, 2012), where higher levels (e.g. greater GM and WM volume) correspond to a better tolerance of age- and disease-related damage (Bartrés-Faz and Arenaza-Urquijo, 2011; Katzman et al., 1988; Mortimer et al., 2003; Stern, 2002, 2012). In contrast, task-based and resting-state fMRI can investigate activation patterns and functional connectivity (Gerchen and Kirsch, 2017). These approaches have been used to investigate mechanisms underlying *‘cognitive reserve’,* referring to the brain’s capacity to functionally adapt or compensate for age- or disease-related neural insult (Bartrés-Faz and Arenaza-Urquijo, 2011; Stern, 2012). Finally, PET and SPECT studies are sensitive to several different biomarkers of Alzheimer’s disease (AD) pathology, including beta amyloid deposition, hypometabolism and perfusion deficits (Scheltens et al., 2016). Leisure activities are widely considered to have *neuroprotective* effects on the aging brain, either by (1) promoting *brain reserve*, (2) supporting *cognitive reserve* or (3) disrupting the progression of AD (Arenaza-Urquijo et al., 2015; Stern, 2002, 2012). Given that the findings of PET and SPECT studies have been comprehensively summarized and discussed elsewhere (Arenaza-Urquijo et al., 2015) and fMRI studies are few in number, this review will primarily focus on structural MRI studies to evaluate the hypothesized role of leisure activities in *brain reserve*.

The neuroanatomical changes that emerge with advanced age have been well characterized by structural MRI studies. Such studies indicate that aging is closely associated with brain atrophy (Raz and Rodrigue, 2006), with GM loss concentrated in the prefrontal cortices and concurrent shrinkage of sub-cortical structures, including the hippocampus (Allen et al., 2005; Apostolova et al., 2012; Driscoll et al., 2009; Gorbach et al., 2017; Raz et al., 2004; Schippling et al., 2017; Taki et al., 2013). Global and region-specific reductions in WM volume are also consistently reported (Allen et al., 2005; Leong et al., 2017; Raz et al., 2004; Schippling et al., 2017) along with the development of periventricular and deep WM hyperintensities in 39–96% of the older adult population (Habes et al., 2016). Diffusion tensor imaging (DTI) studies have provided further insight into the age-related microstructural changes of WM (Madden et al., 2012). Both global and region-specific WM integrity appears to deteriorate with age, as indicated by reductions in fractional anisotropy (FA) and increases in mean diffusivity (MD), radial diffusivity (RD) and axial diffusivity (AD; Bender and Raz, 2015; Bennett and Madden, 2014; Cox et al., 2016; Sexton et al., 2014). Overall, aging is associated with widespread patterns of change in the brain, including GM and WM loss, the appearance of lesions and reduced microstructural quality in the WM, which each contribute to cognitive decline in later life (Bennett and Madden, 2014; Bolandzadeh et al., 2012; Raz and Rodrigue, 2006).

A growing number of studies using MRI techniques have begun to explore the relationship between leisure activities and age-related brain structure (e.g. Foubert-Samier et al., 2012; James et al., 2012; Valenzuela et al., 2008; Vemuri et al., 2016), with mixed evidence regarding whether CA consistently relates to structural MRI markers of aging (Cheng, 2016). As we are not aware of any comprehensive systematic reviews or meta-analyses, we aim to address this gap in the present review. Specifically, our main objective is to systematically summarize the results of cross-sectional and longitudinal MRI studies that have investigated the relationship between levels of CA and SA in older populations. As age affects both GM and WM tissue (Raz and Rodrigue, 2006), we include structural and diffusion-weighted MRI markers in the review to gain insight into whether leisure activities relate to both macro- and micro-structural properties of the aging brain. Based on the rationale that leisure activities are neuroprotective (Arenaza-Urquijo et al., 2015; Bartrés-Faz and Arenaza-Urquijo, 2011; Stern, 2012), we hypothesize that frequently participating in CA and SA will be associated with greater GM and WM volume, higher WM integrity and fewer WM lesions.

## 2 Method

### 2.1 Data sources

Studies were searched for on the online databases EMBASE, MEDLINE and PSYCHINFO, using the interface MEDLINE Ovid in December 2017. The list of terms used in the formal search strategy was compiled after conducting a preliminary exploration of existing literature, where the keywords of articles deemed to be potentially relevant were extracted. An example of the keywords used for the MEDLINE search are shown in Supplementary material: Fig. S1. These terms were adapted for the two remaining databases. The reference lists of relevant studies were also screened to ensure that all literature appropriate to the review was captured and assessed for eligibility.

### 2.2 Inclusion criteria

1 Published as a research article.
2 Engagement in CA and/ or SA was directly assessed with a self-report questionnaire. To maximize the number of studies included in this review, those that had measured both types of activities and combined them for the analyses to produce an overall leisure activity engagement score were also included.
3 The study included a single MRI session (cross-sectional design) or multiple MRI assessments (longitudinal design).
4 An observational study design was used. This criterion was introduced to ensure that the included studies were relatively homogenous in design, allowing for the results to be sufficiently comparable.
5 At least one of the following MRI measures was examined in the study: GM volume, WM volume, WM integrity (e.g. FA, MD) and the presence and severity of WM lesions.
6 The relationship between activity levels and MRI outcomes or group differences in MRI outcomes (i.e. between groups with high/ low activity engagement) was directly examined. Mediation analyses (e.g. examining whether measures of GM volume mediated the association between activity engagement and cognitive function) were not included.
7 The sample consisted of healthy older adults (i.e. without a diagnosis of dementia) with a mean age 60 years or over.
8 The study was published in the English language.

### 2.3 Study identification and selection

Authors MA and ND independently screened the titles and abstracts of all of the citations identified by the search. These authors also reviewed the full text versions of a subset of citations to evaluate whether they met the eligibility criteria for inclusion.

### 2.4 Data extraction

Authors MA and ND separately carried out data extraction using identical structured forms, which were subsequently compared to ensure consistency and accuracy in the information collected. The study-specific information collected included sample characteristics (e.g. age, gender, percentage of females), study design (cross-sectional or longitudinal MRI, duration between measurements of activity engagement and MRI), MRI analyses (e.g. voxel-based morphometry/region-of-interest, WM lesion assessments), activity engagement (e.g. type of activity, life-time period of activity engagement, questionnaire administered), aspects of GM and WM measured (i.e. GM volume, WM volume, WM microstructure, WM lesions) and the findings reported (statistically significant results as indicated by *p* < 0.05, direction of association/group differences observed, co-variates controlled for).

Due to substantial inter-study heterogeneity in the definitions of CA and SA used, we evaluated the set of activities that an individual study had measured against the classifications of CA and SA proposed by Seider et al. (2016), (Supplementary material: Table S1.) and re-categorized them accordingly. For the table of results, studies are divided into either cross-sectional or longitudinal MRI, where the former consists of a single MRI session and the latter integrates multiple MRI assessments. To improve comparability among the reviewed studies, age- and gender-adjusted analyses are reported in the tables of results, where possible. The results of these analyses following further co-variate adjustments (e.g. medical history, body mass index), can be found in the footnotes of each table. If, however, the outcome of age- and gender- controlled analyses had not been included in an article, we instead present their fully adjusted analyses in the tables. Finally, regions found to be significantly associated with activity engagement are grouped based on the major lobe or tract of the brain they are contained within (e.g. frontal, temporal, parietal, occipital and limbic).

To be included in the meta-analysis, studies drawn from the systematic review were required to meet the additional criteria of having performed region-of-interest (ROI) analyses. Voxel-based-morphometry (VBM) studies were excluded as these could introduce substantial bias and are more suitable for alternative methods (e.g. image- or coordinates-based; Salimi-Khorshidi et al., 2009). A meta-analysis was not conducted for local WM volume as only VBM studies had assessed this outcome. Similar to previous publications (Kempton et al., 2008; Sexton et al., 2013), a meta-analysis was only carried out on a particular lobe, subcortical structure or tract if it had been examined by at least three studies. This threshold was not met by studies that had examined white matter microstructure (WMM) or GM volume within the different lobes, hence their results are only considered in the systematic review. Further, we could not directly examine the separate effects of CA and SA due to there being only a small number of studies published to date. The meta-analysis therefore examines the *combined* effects of CA and SA. Since Vemuri et al. (2012) and Vemuri et al. (2016) had overlapping samples, only the former was included in the analysis, as it contributed a larger number of participants.

Comprehensive Meta-Analysis (version 3.3, ^©^ November 20, 2014, Biostat Inc., Englewood, NJ) was used to analyze the data. Sample sizes, p-values and the direction of associations were entered. The program calculated correlation coefficients based on the raw parameters provided, which were transformed into Fisher’s Z before being converted back to correlation coefficients for reporting purposes. Fisher’s r-to-z transformation was used as it provides a less biased summary index when the number of included studies is small (Silver and Dunlap, 1987). Pooled effect sizes are presented as correlation coefficients to improve their interpretability (Borenstein et al., 2009). To calculate these, *random effects models* were used to allow for inter-study variation and generalization to be made beyond the studies included (Borenstein et al., 2010). Cochrane’s Q was used to evaluate inter-study heterogeneity, and publication bias was assessed using funnel plots (presented as Fisher's Z due to the unavailability of plots with correlation coefficients) and Begg and Mazumdar rank correlations test (Begg and Mazumdar, 1994). Authors were also contacted for further information on their analyses to ensure that as many studies as possible were included in this review. If exact p-values could not be obtained, we used the conservative estimate of 0.04 when a p-value within an article had been reported as <0.05 (Sexton et al., 2011). Conversely, a study that reported a non-significant finding as p > 0.05 was estimated as p = 0.5. Only when the direction of a reported association could not be determined, was the study excluded. As a minimum of ten studies are required to perform sub-group analyses and meta-regressions (Higgins and Green, 2011), we were unable to assess the extent to which study design and sample characteristics moderated the examined effects.

### 2.5 Quality assessment

Study quality was evaluated according to the Quality Assessment Tool for Observational Cohort and Cross-sectional Studies (NIH, 2014). This tool consists of fourteen questions, which assess several potential sources of bias in a study. Areas covered include assessment of measure validity, the suitability of the study design to address a given research question, the extent to which the sample is representative of the population of interest and whether key confounders are accounted for in the analyses. Two independent raters (authors MA and ND) rated each study as ‘Good’, ‘Fair’ or ‘Poor’. Any disagreements on the quality rating of a study were resolved through discussion.

## 3 Results

The number of studies yielded at each stage of the search is displayed in Supplementary Material: Fig. S2. A total of eighteen studies were included in this review with the sample characteristics and details of the design of each included study displayed in Table 1.

**Table 1:** Sample characteristicsand design of included studies.

Study	N	Mean Age ± SD	% Female	Design	Activity Assessment	Quality Rating
Arenaza-Urquijo et al. (2016)	45	71.9 ± 6.6	51.1	T1: MRI, CA + SA	25-item CAS (Wilson et al., 2003)	Good
Arfanakis, et al. (2016)	379	82 ± 7	77	T1: MRI, CA + SA	39-item CAS (Wilson et al., 2005)	Good
Bartrés-Faz, et al. (2009)	15	68.3 ± 4.5	73.3	T1: MRI, CA + SA	Study specific questionnaire (Solé-Padullés et al., 2009)	Good
Bennett, et al. (2006)	106	85.2 ± 2.9	48.1	T1: CA, SA; T2: MRI; 1-month interval	Kilsyth Disability Rating Scale (Akhtar et al., 1973)	Good
Foubert-Samier, et al. (2012)	331	76.1 ± 3.9	57.4	T1: MRI, CA, SA	30-item study-specific questionnaire	Good
Gidicsin, et al. (2015)	186	74.4 ± 6	55	T1: MRI, CA + SA	25-item CAS (Wilson et al., 2003)	Good
Gow, et al. (2012)	691	72.7 ± 0.7	47.3	T1: CA + SA; T2: MRI, 3.2 year interval	Study-specific questionnaire	Good
Hafsteinsdottir, et al. (2012)	4304	76.1 ± 5.4^a^	58.5	T1: MRI, CA + SA	10-item study-specific questionnaire	Good
James, et al. (2012)	348	65.2 ± 7.9	0	T1: MRI, SA; T2: MRI, SA; 5 year interval	EFP (Glass, 1998)	Good
Köhncke, et al. (2016)	442	85.1 ± 4.5	68.1	T1: MRI, CA, SA; T2: MRI, CA, SA; 3 year interval	Study-specific questionnaire	Good
Schultz, et al. (2015)	329	60.3 ± 6.3	69	T1: CA + SA; T2: MRI; 1.33 year interval	Modified version of 25-item CAS (Wilson et al., 2003)	Good
Seider, et al. (2016)	65	71.4 ± 8.9	56.9	T1: MRI, CA, SA	CHAMPS (Stewart et al., 1997)	Good
Suo, et al. (2012)	151	80.8 ± 4.6	55.6	T1: MRI; T2: MRI, CA + SA; 2 year interval	LEQ (Valenzuela and Sachdev, 2007)	
Valenzuela, et al. (2008)	37	70.3 ± 5.8	56.9	T1: MRI; T2: MRI, CA + SA; 3 year interval	LEQ (Valenzuela and Sachdev, 2007)	Good
Vaughan, et al. (2014)	393	81.2 ± 4.3	100	T1: MRI, CA + SA	CAS (Wilson et al., 1999)	Good
Vemuri, et al. (2012)	515	79±not reported	43	T1: CA + SA; T2: MRI; 1.5 year interval	Study-specific questionnaire	Good
Vemuri, et al. (2016)	393	78.6 ± 5	38	T1: MRI, CA + SA; T2: MRI; 2.5 year interval	Study-specific questionnaire (Vemuri et al., 2012)	Good
Wirth, et al. (2014)	92	75.2 ± 5.6	58	T1: MRI, CA + SA	25-item CAS (Wilson et al., 2003)	Good

Abbreviations – CA = Cognitive Activity; CA + SA = Composite Measure of Cognitive and Social Activities; CAS = Cognitive Activity Scale; CHAMPS = Community Healthy Activities Model Program for Seniors; EFP = Enacted Function Profile; LEQ = Lifetime of Experiences Questionnaire; MRI = Magnetic Resonance Imaging; N = Number; SA = Social Activity; SD = Standard Deviation; T1 = Time Point 1; T2 = Time Point 2.

a The original study reported sub-group means and SDs, hence, the overall mean age and SD for the sample were calculated (Higgins and Green, 2011). We also present the overall percentage of females in Hafsteinsdottir, et al.’s (2012) study.

### 3.1 Grey matter (GM)

#### 3.1.1 Global GM volume

Five cross-sectional MRI studies have examined the relationship between current CA and SA engagement and global GM volume (Table 2). Three studies (Foubert-Samier et al., 2012; Gow et al., 2012; Vaughan et al., 2014) did not detect any associations between these variables. One study (James et al., 2012) found that global GM volume positively correlated with SA levels, but found no association between changes in total GM volume and activity engagement at 5-year follow-up. In group comparisons, adults who most frequently participated in socially- and cognitively-demanding activities exhibited greater global GM volume when compared to those who were the least active in the cohort (Hafsteinsdottir et al., 2012).

**Table 2:** Cross-sectional MRI studies that have investigated the association between CA and SA engagement levels and global GM volume.

Study	Period of Life	Activity	Results	Co-variates
Foubert-Samier et al. (2012)	Mid-life Current Mid-life Current	CA^a^ CA SA SA	n.s n.s. n.s. n.s.	Age, gender, education, APOE4
Gow et al. (2012)	Current	CA + SA	n.s.	Age, gender
Hafsteinsdottir et al. (2012)	Current	CA + SA	↑***, b	Age, gender, education, BMI, CHD, hypertension, MCI, diabetes, smoking status
James et al. (2012)^c^	Current	SA	↑	Age, education, ICV, ethnicity/ race, diabetes, hypertension, handedness, group (former lead workers/ controls)
Vaughan et al. (2014)	Current	CA + SA	n.s.	ICV

Abbreviations- APOE4 = Apolipoprotein E ε4; BMI = Body Mass Index; CA = Cognitive Activity; CA + SA = Composite Measure of Cognitive and Social Activities; CHD = Coronary Heart Disease; ICV = Intracranial Volume; MCI = Mild Cognitive Impairment; ROI = Region-of-Interest; SA = Social Activity; VBM = Voxel-based Morphometry; ↑ refers to a positive relationship between activity engagement and whole-brain measures of GM volume.

a These authors categorized mid-life and current CA into those that were cognitively stimulating and those that were not. None of these combinations of variables (i.e. mid-life/ current, stimulating/non-stimulating CA) resulted in significant associations with whole-brain GM volume.

b Hafsteinsdottir et al. (2012) sub-divided their sample into 4 groups, to represent different levels of leisure activity engagement. Here, ↑ demonstrates the finding that participants in the highest quartile exhibited significantly greater global GM, compared to those in the lowest quartile.

c While James et al. (2012) employed a longitudinal MRI design, their primary analysis was cross-sectional, hence, these results are displayed in the above table. The outcome of their secondary analyses, which investigated associations between activity engagement and global GM volume across a 5-year interval are considered, in the main text.

*** p < 0.0001.

**Fig. 1:**
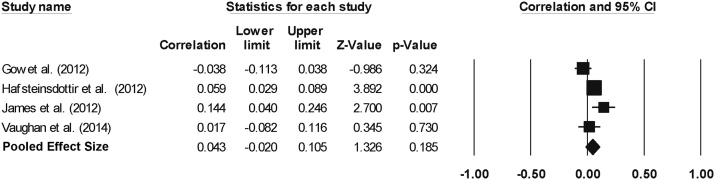
Effect sizes (correlation coefficient) for total GM volume: No significant association between CA and SA engagement and global GM volume.

A meta-analysis was conducted on four of these studies (pooled n = 5718), which revealed that there was no association between late-life leisure activity levels and global GM volume (p = 0.185, Fig. 1). Significant inter-study heterogeneity was, however, detected (Q = 9.123, p = 0.028, I^2^ = 67.117). Visual inspection of a funnel plot (Supplementary material: Fig. S3) and a non-significant result from the Begg and Mazumdar rank correlation test (τ = 0.167, two-tailed p = 0.734) suggested that there was no substantial publication bias.

#### 3.1.2 Local GM volume

Table 3 presents the results of the twelve studies that have examined the relationship between non-physical activity participation and local GM volume. There were inter-study variations in the type of MRI analysis employed (i.e. VBM or ROI). Several of the reviewed ROIs studies reported associations between SA and CA engagement and GM volume that spanned all major lobes of the brain (James et al., 2012; Schultz et al., 2015; Seider et al., 2016). Among the cross-sectional VBM studies identified, late-life CA and SA participation have also been linked to GM volume in the frontal, parietal, temporal and occipital lobes (Arenaza-Urquijo et al., 2016; Bartrés-Faz et al., 2009). In their voxel-wise analyses, James et al. (2012) additionally found that more frequent current participation in SA correlated with greater temporal and parietal GM volume. One cross-sectional VBM study (Foubert-Samier et al., 2012), however, did not report any significant relationships between local GM volume and CA and SA participation during either mid-life or late-life.

**Table 3:** Cross-sectional and longitudinal MRI studies that have investigated the relationship between CA and SA participation and local GM volume.

Study	MRI Analysis	Period of Life	Activity Type	Results		Significant Lobes (Regions)	Co-variates
*Cross-sectional Studies*							
Arenaza-Urquijo et al. (2016)	VBM	Current	CA + SA		↑**	Frontal (middle frontal gyrus), Parietal, Temporal (parahippocampal gyrus, inferior temporal gyrus, superior temporal gyrus, temporal pole) Occipital (angular gyrus), Limbic (caudate, insular cortex)	Age, gender^a^
Bartrés-Faz et al. (2009)	VBM	Life-time	CA + SA		↑	Frontal (superior frontal gyrus, medial frontal gyrus); Parietal (supramarginalis gyrus)	Age, gender, MMSE
Bennett et al. (2006)	ROI	Current	CA (reading) SA (socializing)		n.s. ↑	Limbic (hippocampus)	Age, gender education
Foubert-Samier et al. (2012)	VBM	Mid-life Current Mid-life Current	CA^b^ CA SA SA		n.s. n.s. n.s. n.s.		Age, gender, education, APOE4, laterality, ICV
Gidicsin et al. (2015)	ROI	Current	CA + SA		n.s.		Age, education, APOE4, NART IQ, past CA, pedometer assessed total walking speed
James et al. (2012)	ROI VBM	Current Current	SA SA		↑ ↑	Temporal, Occipital Temporal, Parietal	Age, education, ethnicity/ race, diabetes, hypertension, handedness, group (former lead workers/ controls), ICV
Schultz et al. (2015)	ROI	Current	SA (games) CA + SA		↑ n.s.	Frontal (middle frontal gyrus), Limbic (posterior and anterior cingulate)	Age, gender, time interval between CAS and MRI, ICV
Seider et al. (2016)	ROI	Current	CA		↑	Frontal, Parietal, Temporal, Occipital, Limbic (thalamus, caudate, hippocampus, amygdala)	Education
			SA		n.s.		
Suo et al. (2012)	VBM	Early-life Mid-life Current	CA + SA CA + SA CA + SA		n.s. ↑^c^ n.s	Limbic (hippocampus)	Age, gender, cardiovascular risk factor scale, PA, ICV
			CA + SA				
Vemuri et al. (2012)	ROI	Current	CA + SA		n.s.		Age, gender
*Longitudinal Studies*							
Valenzuela et al. (2008)	ROI	Life-time Life-time	CA + SA^c^ CA + SA	↑ ↓		Limbic (hippocampus) Limbic (hippocampal atrophy)	Age, gender, hypertension, ICV
		Mid-life	CA + SA	↓		Limbic (hippocampal atrophy)	
		Late-life	CA + SA	↓		Limbic (hippocampal atrophy)	
Vemuri et al. (2016)	ROI	Mid-life	CA + SA	n.s.			Age, gender, education, occupation, APOE4, mid-life PA

Abbreviation –APOE4 = Apolipoprotein E ε4; CA = Cognitive Activity; CA + SA = Composite Measure of Cognitive and Social Activities; GM = Grey Matter; ICV = Intracranial volume; MMSE = Mini Mental State Examination; MRI = Magnetic Resonance Imaging; NART = National Adult Reading Test; n.s. = not significant; PA = Physical Activity; ROI = Region-of-Interest; SA = Social Activity; VBM = Voxel Based Morphometry; ↑ refers to a positive relationship between activity engagement and local measures of GM volume. ↓ indicates an inverse association between participation in leisure activities and hippocampal atrophy.

** *p* < 0.001.

a After additionally co-varying for late-life PA, the following regions remained significantly related to CA: Frontal (middle frontal gyrus) Parietal (precuneus cortex), Temporal (parahippocampal gyrus, temporal pole), Occipital (angular gyrus) Limbic (caudate, insular cortex).

b In this study, mid-life and current CA were examined separately, which were further separated into those that were cognitively stimulating and those that were not. There were no significant associations between any combinations of variables (i.e. mid-life/ current, stimulating/non-stimulating CA) and local GM volume.

c For Suo et al., ↑ demonstrates that participants with high mid-life LEQ scores (i.e. a composite score reflecting high educational and occupational attainment and frequent activity engagement) exhibited greater GM in the hippocampus, compared to participants with low mid-life LEQ scores.

The subcortical structure most commonly assessed was the hippocampus. While null findings have been reported (Gidicsin et al., 2015; Schultz et al., 2015; Vemuri et al., 2012, 2016), hippocampal volume has been found to correlate positively with regular late-life engagement in CA (Seider et al., 2016), SA (Bennett et al., 2006; James et al., 2012) or both types of activities (Bennett et al., 2006; Valenzuela et al., 2008) in a number of studies. Suo et al. (2012) found that *mid-life, but not current*, activity levels were associated with GM in the hippocampus. Further, Valenzuela et al., (2008) reported that participants who regularly undertook leisure activities over their life-time exhibited less decline in hippocampal volume over three years (3.6%), compared to inactive individuals (8.3%).

**Fig. 2:**
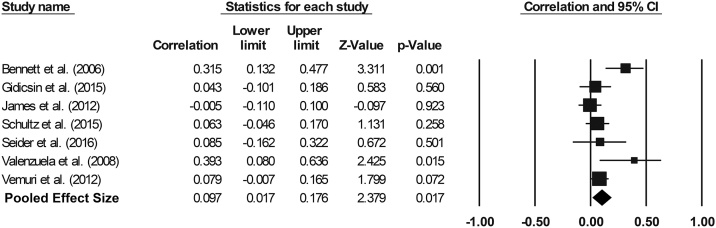
Effect sizes (correlation coefficient) for hippocampal volume: Higher levels of CA and SA engagement are associated with greater hippocampal volume.

A meta-analysis was conducted using seven of the abovementioned ROI studies that had examined the hippocampus (pooled n = 1455), to reveal a small, yet significant effect size of 0.097 (95% confidence interval = 0.017 to 0.176, p = 0.017, Fig. 2). Significant inter-study heterogeneity was also detected (Q = 12.919, p = 0.044, I^2^ = 53.557). Publication bias appeared to be absent, as shown by a funnel plot (Supplementary material: Fig. S4) and a non-significant Begg & Mazumdar rank correlation test (τ = 0.286, two-tailed p = 0.368).

### 3.2 White matter (WM)

#### 3.2.1 Global WM volume

Six studies have investigated the cross-sectional relationship between late-life participation in SA and CA and global assessments of WM volume. As shown in Table 4, the results of four of these studies (Bennett et al., 2006; James et al., 2012; Foubert-Samier et al., 2012; Vaughan et al., 2014) were not significant, with one study also failing to find an association with mid-life CA or SA levels (Foubert-Samier et al., 2012). When controlling for age and gender, Gow et al. (2012) reported that non-physical leisure activities in late adulthood were positively associated with normal appearing WM volume across the brain. However, this association was no longer significant after accounting for additional co-variates, including childhood IQ, occupation and medical history. The largest study published in this area to date (n = 4304; Hafsteinsdottir et al., 2012), found that older adults who were most regularly engaged in social and intellectually stimulating activities had higher levels of total WM volume, compared to individuals who were the least engaged.

**Table 4:** Results of cross-sectional MRI studies that have examined the association between CA and SA participation and global WM volume.

Study	MRI Analysis	Period of Life	Activity Type	Results	Co-variates
Bennett et al. (2006)	ROI	Current	CA (reading) SA (socializing)	n.s. n.s.	Age, gender, education
Foubert-Samier et al. (2012)	VBM	Mid-life Current Mid-life Current	CA^a^ CA SA SA	n.s. n.s. n.s. n.s.	Age, gender, APOE4, laterality, ICV
Gow et al. (2012)	ROI	Current	CA + SA	↑	Age and gender^a^
Hafsteinsdottir et al. (2012)	ROI	Current	CA + SA	↑***^,b^	Age, gender, education, BMI,CHD, hypertension, MCI, diabetes, smoking status
James et al. (2012)	VBM	Current	SA	n.s.	Age, education, ICV, ethnicity/race, diabetes, hypertension, handedness, group (former lead workers/ controls)
Vaughan et al. (2014)	ROI	Current	CA + SA	n.s.	ICV

Abbreviations – APOE4 = Apolipoprotein E ε4; BMI = Body Mass Index; CA = Cognitive Activity; CA + SA = Composite Measure of Cognitive and Social Activities; CHD = Coronary Heart Disease; SA = Social Activity; ICV = Intracranial volume; MCI = Mild Cognitive Impairments; n.s. = not significant; ↑ demonstrates that increasing levels of activity engagement are associated with greater global measures of WM volume.

^a^Foubert-Samier et al. (2012) evaluated mid-life and current CA individually, and divided CA into either cognitively stimulating or not stimulating activities. There were no significant associations between any combinations of variables (i.e. mid-life/ current, stimulating/non-stimulating CA) and whole-brain WM volume.

^a^After additional adjustments were made for IQ at the age of 11 and social class, this association became non-significant.

^b^These authors divided the cohort into quartiles according to overall leisure activity engagement levels. In this instance, ↑ indicates that participants in the lowest quartile of activity engagement had significantly smaller WM volume, compared to participants who were in the highest active quartile.

*** *p* < 0.0005.

**Fig. 3:**
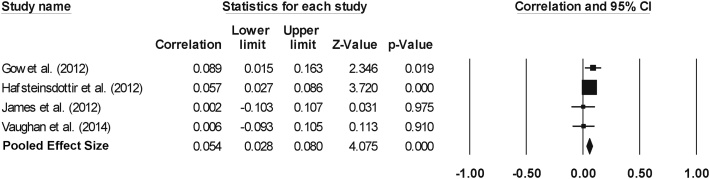
Effect sizes (correlation coefficient) for total WM volume: a positive relationship between CA and SA levels and whole-brain WM volume.

A meta-analysis of four studies (pooled n = 5736) revealed a significant and small effect size of 0.054 between current activity levels and global WM volume (95% confidence interval = 0.028 to 0.080, p < 0.001, Fig. 3). Tests for heterogeneity (Q = 2.75, p = 0.432, I^2^ = 0.000) and publication bias, as assessed by a funnel plot (Supplementary material: Fig. S5) and the Begg & Mazumdar rank correlation test, were not significant (τ = 0.500, two-tailed p = 0.308).

#### 3.2.2 Local WM volume

Two studies examined regional measures of WM volume in relation to activity engagement (Table 5). While one of these studies reported null findings for mid-life and current activity levels (Foubert-Samier et al., 2012), the second study identified a positive cross-sectional relationship between current social activity participation and WM volume of the corpus callosum (James et al., 2012).

**Table 5:** Findings of cross-sectional MRI studies that have addressed the relationship between CA and SA engagement and region-specific measures of WM volume.

Study	MRI Analysis	Period of Life	Activity Type	Results	Significant Regions	Co-variates
Foubert-Samier et al. (2012)	VBM	Mid-life Current Mid-life Current	CA^a^ CA SA SA	n.s. n.s. n.s. n.s.		Age, gender, APOE4, laterality, ICV
James et al. (2012)	VBM	Current	SA	↑	corpus callosum	Age, education, ICV, ethnicity/ race, diabetes, hypertension, handedness, group (former lead workers/ controls)

Abbreviations – APOE4 = Apolipoprotein E ε4; CA = Cognitive Activity; CA + SA = Composite Measure of Cognitive and Social Activities; SA = Social Activity; ICV = Intracranial volume; n.s. = not significant; ↑ demonstrates that increasing levels of SA engagement are associated with greater levels of local WM volume.

a CA engagement was not only divided into mid-life and late-life but also further separated into sub-categories stimulating and not stimulating. No associations between any combination of variables were significant (i.e. mid-life/late-life, stimulating/ not-stimulating CA) and local WM.

#### 3.2.3 Global WM lesions

In total, seven studies have investigated the relationship between activity participation and whole-brain assessments of WM lesions (Table 6). One study found a positive cross-sectional correlation between life-time CA and SA engagement and global WM lesion volume, and further analyses indicated that mid-life and current activity levels were individually related to this outcome, although activity levels accumulated over a life-time was not (Wirth et al., 2014). Another study suggested that participants in the highest activity engagement quartile had lower volumes of WM hyperintensities (a difference of 12%), when compared to the least active quartile (Hafsteinsdottir et al., 2012). A further study did not detect any cross-sectional associations between activity engagement and WM lesions (Köhncke et al., 2016). It did, however, find that declines in activity engagement over a three-year period were related to longitudinal increases in whole-brain WM lesions volume (Köhncke et al., 2016). Four studies reported a lack of association between current or life-time levels of activity participation, WM lesion volume or severity rating (Bennett et al., 2006; Gow et al., 2012; Valenzuela et al., 2007; Vaughan et al., 2014).

**Table 6:** Findings of cross-sectional and longitudinal studies that have investigated whether CA and SA engagement is related to whole-brain WM lesions.

Study	WM lesions Outcome	Period of Life	Activity Type	Results	Co-variates
*Cross-sectional Studies*					
Bennett et al. (2006)	Periventricular WM lesions	Current	CA (reading) SA (socializing)	n.s. n.s.	Age, gender and education
Hafsteinsdottir et al. (2012)	WM lesion volume	Current	CA + SA	↓**, b	Age, gender, education, BMI CHD, hypertension, MCI, diabetes, smoking status
Gow et al. (2012)	WM lesion volume (%ICV) WM lesion severity^a^	Current Current	CA + SA CA + SA	n.s. n.s.	Age and gender
Vaughan et al. (2014)	WM lesion volume	Current	CA + SA	n.s.	ICV
Wirth et al. (2014)	WM lesion volume (ICV adjusted)	Early-life Mid-life Current Life-time	CA + SA CA + SA CA + SA CA + SA	↓ ↓ ↓ n.s.	Age, gender and education
*Longitudinal Studies*					
Köhncke et al. (2016)	WM lesion volume	Current	CA (baseline) CA (change) SA (baseline) SA (change)	n.s. n.s. n.s; ↓	Age, gender and education
Valenzuela et al. (2008)	WM lesion volume	Life-time	CA + SA	n.s.	Age, gender, hypertension and ICV

Abbreviations – BMI = Body Mass Index; CA = Cognitive Activity; CA + SA = Composite Measure of Cognitive and Social Activities; CHD = Coronary Heart Disease; SA = Social Activity; ICV = Intracranial volume; MCI = Mild Cognitive Impairments; n.s. = not significant; ↓ demonstrates that increasing levels of activity engagement are associated with lower levels of global WM lesions.

a Fazekas scale was used to rate WM lesions identified in FLAIR and T2-weighted images as either periventricular or deep lesions, separately for both hemispheres. An overall WM lesion score was then calculated for each participant, using these ratings.

b For this study, ↓ indicates that participants in the highest activity engagement quartile had significantly greater levels of global WM lesions, relative to individuals in the lowest quartile group.

** *p* < 0.005.

**Fig. 4:**
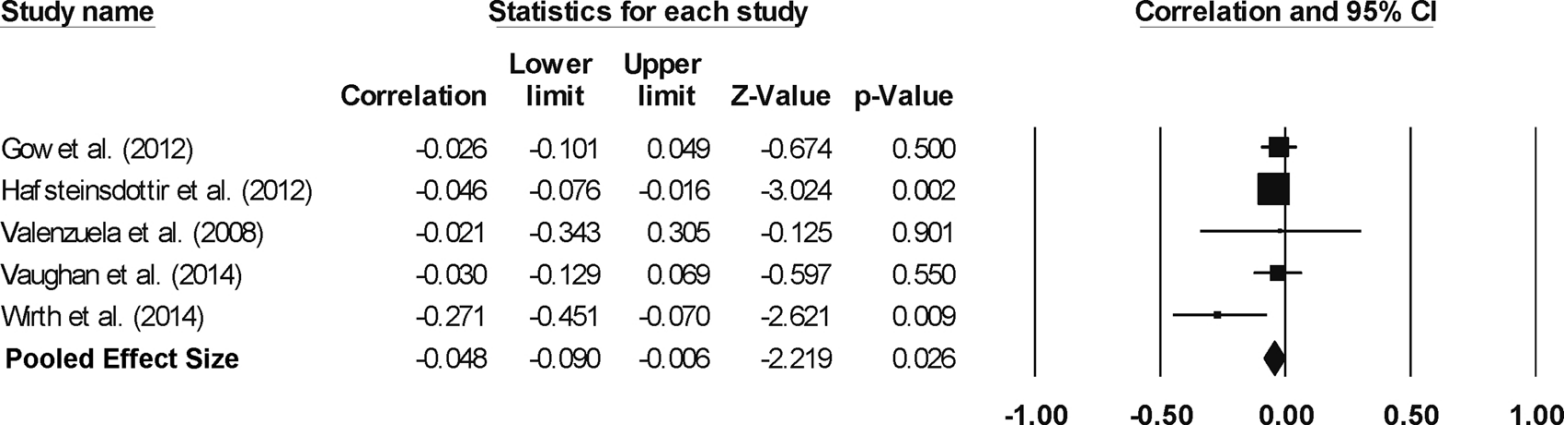
Effect sizes (correlation coefficient) for global WM lesions: Higher CA and SA levels are related to a reduction in whole-brain WM lesion volume.

A meta-analysis of five of these studies (pooled n = 5587) detected a significant effect size of −0.048 (95% confidence interval = −0.090 to −0.006, p = 0.026, Fig. 4). The test for heterogeneity was not significant (Q = 5.020, p = 0.285, I^2^ = 20.314). Further, inspection of the funnel plot (Supplementary material: Fig. S6) and a non-significant Begg and Mazumdar rank correlation test (τ = −0.100, two-tailed p = 0.308p = 0.806) suggested an absence of publication bias.

#### 3.2.4 WMM

Three studies (2 cross-sectional, 1 longitudinal; Table 7) employed DTI to investigate associations between activity engagement and WMM. One study reported that higher levels of current CA and SA was related to greater FA in tracts including the left superior and inferior longitudinal fasciculi, fornix and corpus callosum (Arfanakis et al., 2016), and smaller AD and RD in a cluster localized to the thalamus. The second study (Köhncke et al., 2016) found that increasing SA engagement over a three-year period was associated with greater FA and smaller MD in the corticospinal tract, with no significant associations to RD or AD in this region. Additionally, increasing participation in low complexity activities (e.g. television watching) was related to greater MD within this tract. Neither CA nor SA significantly correlated with DTI measures in any other of the WM tracts assessed, which consisted of the superior longitudinal fasciculus, forceps major and minor and cingulum cingulate gyrus (Köhncke et al., 2016). The third study did not find a relationship between a composite measure of late-life SA and CA participation and global WMM (Gow et al., 2012).

**Table 7:** Results of cross-sectional and longitudinal DTI studies to have examined associations between CA and SA participation and WMM.

Study	DTI Analysis	Period of Life	Activity Type	Results	Significant Regions	Co-variates
*Cross-sectional Studies*						
Arfanakis, et al. (2016)	TBSS	Current Current	CA + SA CA + SA	↑FA ↓AD, ↓TD, ↓RD	superior and inferior longitudinal fasciculi, fornix, corpus callosum thalamus	Age, gender, education, early-life CA resources, WM lesions
Gow et al. (2012)	Tractography (12 WM tracts)	Current	CA + SA	FA n.s.		Age, gender
*Longitudinal Studies*						
Köhncke et al. (2016)	TBSS	Current Current Current Current	CA (baseline)^a^ CA (change) SA (baseline) SA (change)	FA, MD (baseline) n.s. FA, MD (change) n.s. FA, MD (baseline) n.s. ↑FA, ↓MD (change)*	corticospinal	Age, gender, education

Abbreviations- AD = Axial Diffusivity; CA = Cognitive Activity; CA + SA = Composite Measure of Cognitive and Social Activities; DTI = Diffusion Tensor Imaging; FA = Fractional Anisotropy; MD = Medial Diffusivity; n.s. = not significant; RD Radial Diffusivity; SA = Social Activity; TBSS = Tract-Based Spatial Statistics; TD = Trace Diffusivity; WM = White Matter.

a These authors sub-categorized CA activities as complex (e.g. travelling) or low-level (e.g. watching television). Following this, they used a latent change model to examine the cross-sectional (baseline) and change-change associations between each of these types of activities and FA and MD values. The only significant finding reported was a positive change-change relationship between low complexity CA and MD values.

* *p* < 0.01.

## 4 Discussion

### 4.1 Summary of findings

Prompted by epidemiological findings implicating a link between CA and SA levels and cognition in old age, an increasing number of MRI studies have investigated whether socio-intellectual activities directly relate to the structural integrity of the older adult’s brain. We aimed to summarize the findings of these MRI studies in the present systematic review and meta-analysis. Through our literature search, we identified eighteen studies that met the eligibility criteria of this review. Overall, the reviewed evidence suggests that composite measures of CA and SA correlate with whole-brain volumetric assessments of WM volume and lesions, in addition to regional GM volume across the frontal, parietal, temporal, occipital and limbic lobes, and the hippocampus. Despite the significant regional GM findings, there was no association found between activity levels and global GM volume. Furthermore, the results regarding WMM were inconclusive. Due to an insufficient number of studies published and mixed findings among existing studies, we are unable to make inferences concerning the independent associations of CA and SA with GM or WM outcomes. Importantly, for most of the associations identified in the reviewed literature, significant as well as null findings were reported. However, we regard the current evidence as promising and hold the view that future studies will help to further delineate the extent to which CA and SA levels relate to the aging brain. We next discuss the implications of the reviewed findings for the hypothesized role of leisure activities in brain aging, the potential neurobiological mechanisms mediating these effects, methodological considerations and avenues for further work.

### 4.2 Do socio-intellectual activities support neural mechanisms?

GM and WM indices are often considered proxies of *brain reserve* (Stern, 2002, 2012) among neuroimaging studies (Bartrés-Faz and Arenaza-Urquijo, 2011). The associations found between leisure activities and GM and WM integrity may therefore suggest that *brain reserve* is a potential mechanism underlying the link between socio-intellectual activities and old age cognition. This is unlikely to be the only mechanism at play, as fMRI studies examining leisure activities also suggest a role of *cognitive reserve* in promoting cognitive function, in both healthy aging and AD (Arenaza-Urquijo et al., 2015; Bartrés-Faz et al., 2009; McDonough et al., 2015). For example, teaching a group of older individuals challenging and novel activities (i.e. digital photography and quilting) over a period of 14 weeks has been reported to improve the modulation of neural activity while performing semantic judgment tasks (McDonough et al., 2015). Being socially and intellectually engaged also appears to relate to reduced resting-state functional connectivity in the right inferior frontal cortex, interpreted as higher neural efficiency (Bartrés-Faz et al., 2009). A comprehensive review by Arenaza-Urquijo et al. (2015) further suggests that leisure activities may directly interact with the development of AD biomarkers (e.g. β-amyloid accumulation (Aβ), hypometabolism and brain atrophy) amongst healthy older adults. Indeed, higher lifetime engagement in cognitive activities has been related to lower levels of cortical Aβ as measured by [(11)C] labeled Pittsburgh-Compound-B PET imaging (Landau et al., 2012; Wirth et al., 2014). Taken together, there are at least three potential mechanisms underlying the associations found between leisure activities and old age cognition: *brain reserve, cognitive reserve* and slowing down the development of AD-related pathology. While our review suggests a role for *brain reserve*, it is yet to be determined whether these mechanisms are concurrently engaged, or whether one overtakes the other as an individual ages. Evidently, longitudinal imaging studies combining both structural and functional imaging techniques are needed to address this key outstanding question.

### 4.3 Neurobiological mechanisms

**Fig. 5:**
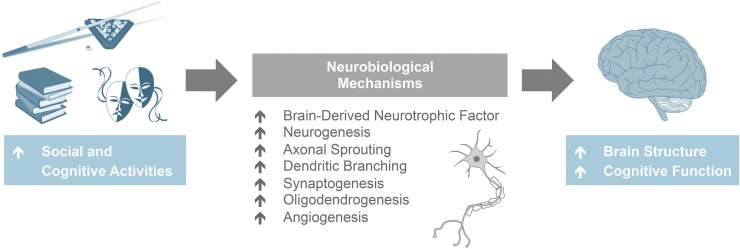
Schematic diagram outlining the proposed neurobiological mechanisms that may support associations between activities and brain reserve and cognition, in old age.

Several potential candidate processes may support the associations found between socio-intellectual activities and neuroimaging markers of *brain reserve* (overview provided in Fig. 5). For example, regularly engaging in socially- and intellectually- stimulating activities could increase the up-regulation of brain-derived neurotrophic factor (BDNF), a molecule that stimulates neuronal growth and cell proliferation and is known to decline with age (Erickson et al., 2010). Sustained periods of activity might promote higher levels of BDNF, potentially contributing to neurogenesis or prolonging the survival of cells (Valenzuela et al., 2007). Indeed, greater neuronal density in the prefrontal cortex has been found in relation to more cognitively active lifestyles, at autopsy (Valenzuela et al., 2012). Synaptic or dendritic plasticity might also be enhanced, as indicated by increases in the number of synapses of neurons in the rat visual cortex following housing in a complex environment for a month (Briones et al., 2004). Dendritic spines in the somatosensory cortex have also been shown to be sensitive to environmental enrichment in mice (Jung and Herms, 2014).

BDNF additionally appears to preserve the integrity of WM (Maillard et al., 2016) and promotes recovery following WM damage (Mamounas et al., 2000). This is attributed to the regulatory role of BDNF in the regenerating oligodendrocyte progenitor cells (OPCs), which are essential for the continued renewal of myelin in the central nervous system (Miyamoto et al., 2015; Van’t Veer et al., 2009) with the production of OPCs typically attenuated with aging (Miyamoto et al., 2013). Interestingly, exposing adult rats to an enriched environment seems to lead to enhanced numbers of OPCs in the amygdala (Okuda et al., 2009) and corpus callosum (Zhao et al., 2011) relative to non-enriched rats. Although the evidence is limited, the forming of new blood vessels may explain the emerging protective role of CA and SA against WM lesions (Valenzuela et al., 2007), which develop as a consequence of long-term ischaemia and hypoxia (Prins and Scheltens, 2015). Overall, there are a number of potential mechanisms that may support the reported links between activity and brain structure, including neurogenesis, synaptogenesis, oligodendrogenesis and angiogenesis.

The following sections provide an overview of the current state of the reviewed evidence. We identified four methodological themes within the literature and these are each addressed in turn.

### 4.4 Methodological considerations: demographics and generalizability

There was substantial heterogeneity within the pool of studies examined, including in their designs and sample characteristics. Nevertheless, all studies received a ‘Good’ rating suggesting that the quality of the evidence reviewed was highly consistent. We also generally found that participant demographics and recruitment methods were sufficiently described. Most of the included studies were based on secondary analyses of existing cohort data, where typically a subset of the baseline sample was used. Some studies used less than 10% of the original sample (e.g. 3.3%, Köhncke et al., 2016; 8.94%, Vemuri et al., 2016). A caveat of focusing on a such a small sub-group of participants is that it may inadvertently introduce a ‘healthy volunteer’ bias (Delgado-Rodriguez, 2004), which was indeed observed for studies reporting the characteristics of included and excluded participants. Specifically, participants included in the analytical sample were generally younger, more cognitively intact (as indicated by MMSE scores) and less likely to be diagnosed with hypertension or diabetes (Foubert-Samier et al., 2012; Hafsteinsdottir et al., 2012). A portion of the reviewed studies also focused on select samples of older adults, such as male ex-employees of a lead manufacturing company (James et al., 2012) or older women (Vaughan et al., 2014). As a consequence, the results reported by these studies are unlikely to generalize to the wider older adult population. The present review was, however, unable to formally assess the existence of moderating variables using meta-regression due to the small number of studies included. Nevertheless, future reviews will be able to statistically evaluate whether factors, such as gender or MMSE score, moderate the reported associations as more data becomes available. This will help to clarify whether the review findings do indeed generalize to older adults as a whole, or are most relevant for specific sub-groups.

### 4.5 Methodological considerations: study design and directionality

Cross-sectional MRI was the predominant design employed among the included studies, with only a small number integrating repeat MRI measurements (James et al., 2012; Köhncke et al., 2016; Suo et al., 2012; Valenzuela et al., 2008; Vemuri et al., 2016). The reviewed evidence was also correlational in nature, suggesting that the hypothesis of leisure activities impacting upon brain structure is not the only way to account for the present findings. For example, our results could be explained by progressive brain atrophy causing individuals to withdraw from hobbies, and/or those with larger brains being more inclined to participate in an intensive routine of activities. Alternatively, a common third variable (e.g. age) might underlie the associations reported. It should be noted that almost all of the included studies attempted to minimize this risk. For example, age and gender were typically controlled for in analyses, with further adjustments made for other co-variates (e.g. education, Apolipoprotein ε4 genotype). Nevertheless, the findings of well-designed RCTs need to be taken into consideration, so as to thoroughly evaluate whether a causal and independent relationship exists between leisure activities and late-life brain structure.

A growing number of RCTs have begun to address the question of causality. Many of these studies indicate that GM enlargements and WM microstructural changes occur after a period of engagement in various mentally challenging tasks, including learning novel color names (Kwok et al., 2011), 3-ball juggling (Driemeyer et al., 2008; Scholz et al., 2009) and training in memory (de Lange et al., 2016) and reasoning domains (Mackey et al., 2012). More generally, cognitive training has reportedly lead to both structural and functional neural changes in older adults (reviewed in Ten Brinke et al., 2017). Additionally, socializing at a community center over the course of 40 weeks appears to have increased total brain volume within a sample of elderly adults (Mortimer et al., 2012). Overall, published RCTs provide further support towards the hypothesis that leisure activities directly affect brain structure. These studies are, however, not without their shortcomings. Many RCTs are currently limited by their short follow-up periods and small sample sizes, highlighting the need for large-scale longitudinal studies investigating the effects of leisure activities on the aging brain.

### 4.6 Methodological considerations: measurements of leisure activities and risk of bias

A key consideration for our review is the risk of measurement error attached to self-report questionnaires, which can be somewhat minimized if the measures used are validated. We generally found that assessments of leisure activities had acceptable psychometric properties. On the other hand, we also came across studies that had employed activity measures without evaluating their validity or reliability. These studies were, however, few in number. The most commonly employed questionnaire was the Cognitive Activities Scale (CAS; Wilson, 1999, 2003, 2005), which is widely validated and has been established in a research setting. This measure collects information on late-life activity levels, in addition to retrospectively asking respondents about activity engagement across the lifespan (e.g. childhood, early- and mid-adulthood). The CAS is clearly advantageous, as it can be used to probe how activity levels at different lifetime periods relate to various neuroimaging metrics of interest. On the other hand, it is also susceptible to misreporting, particularly among older adults with memory-related impairments (Gow et al., 2017). This limitation may be avoidable if researchers prioritize cohort studies that have collected longitudinal data on activity levels and neuroimaging outcomes (Gow et al., 2017).

Despite efforts to ensure that the questionnaires used in research are psychometrically sound, they remain susceptible to reporting biases including social desirability (Adams et al., 2005) and problems with memory recall (Gow et al., 2017). Unfortunately, there is a scarcity of established objective measures of SA and CA that could easily overcome these problems inherent to self-report instruments. One potential solution may be to employ validated objective assessments used for other health behaviors (e.g. diet, physical activity), within this area of research. We suggest that wearable technology represents a feasible candidate that may be adopted by studies focusing on socio-intellectual activities in the near future.

A wearable device often takes the form of a small portable camera worn around the neck or a badge worn on shirts, with the main function being to manually or automatically capture photos of the wearer’s environment (Bai et al., 2012; Hodges et al., 2006; Sun et al., 2014). This type of technology has been used as an aid for individuals with memory impairments (Hodges et al., 2006), and more recently, as a research tool for the assessment of several modifiable behaviours, including diet and physical activity (Sun et al., 2014). One example of a wearable device is the SenseCam, which has been shown to collect data more accurately (e.g. calorie intake) than detailed self-report assessments (e.g. food diaries; O’Loughlin et al., 2013). Perhaps most importantly for the present review, this device can be used to differentiate between activities of varying social demands, such as those that are conducted alone or within a public space (with minimal interactions, such as within a shopping center) or that feature direct face-to-face interactions (Doherty et al., 2013). Overall, the described findings suggest that wearable technology may offer an objective way to measure socio-intellectual activities, which may be advantageous over currently used self-report methods.

### 4.7 Methodological considerations: defining and distinguishing between cognitive and social activities

The final, but no less important, methodological theme concerns the definition and operationalization of CA and SA. Generally, CA and SA were not explicitly defined within the reviewed evidence, representing an issue observed more broadly across neuroimaging and epidemiological studies (Cheng, 2016; Hertzog et al., 2009). There were, however, a few exceptions (e.g. Seider et al., 2016). It might be argued that these constructs are self-explanatory and generally agreed upon, hence, no explicit definitions are required. Yet, this did not appear to be the case for the reviewed evidence, as indicated by considerable differences between studies in the number and type of individual activities used to define CA and SA constructs. Furthermore, activities that involved socializing (e.g. playing games) were sometimes classed as ‘social’ (Köhncke et al., 2016; Seider et al., 2016) and other times as ‘cognitive’ (Vemuri et al., 2012, 2016). To address these inter-study inconsistencies, we next outline potential criteria for CA and SA, which have been developed from existing definitions proposed in the broader literature. Authors of future studies could refer to these criteria when deciding on whether a given activity should be categorized as socially or cognitively demanding.

Broadly speaking, previous definitions of CA have mainly emphasized a key role of information processing (Wilson et al., 2005; Wilson and Bennett, 2003), or otherwise necessitated that an activity is mentally challenging or stimulating (Hertzog et al., 2009; Seider et al., 2016; Yates et al., 2016). What constitutes mental challenge/ stimulation is, however, often left unspecified. The novelty of the information encountered could potentially be considered a defining feature of cognitive complexity (e.g. when learning a language; Hultsch et al., 1999; Jopp and Hertzog, 2010). Yet, novelty alone is not sufficient. For example, a viewer may come across several new facts while watching the television, but only passively take this information in (Cheng, 2016). The *passive* nature of this activity may even partly account for why television watching is emerging as a potentially *harmful* activity for cognition (e.g. Hoang et al., 2016), relative to traditional ‘cognitive’ activities (e.g. reading; Arfanakis et al., 2016). This would suggest that *active* engagement with the information encountered is a crucial component for CA. In other words, it should involve the recruitment of one or several higher-level cognitive domains (e.g. executive function, memory; Hughes, 2010; Jopp and Hertzog, 2010). Cognitive training paradigms are indeed based on the premise that cognitive domains can be enhanced through intensive and repeated practice of them (Jak et al., 2013). Overall, it appears that ‘mentally challenging/ stimulating’ activity (i.e. CA) should: (1) involve a degree of novelty (e.g. solving a new crossword puzzle, reading a book on an unfamiliar topic) and (2) necessitate one or more higher-level cognitive functions in order to successfully complete it.

SA are typically defined as activities undertaken with others, with solitary activities on the other end of the spectrum (e.g. Seider et al., 2016; Singh-Manoux et al., 2003). Although the presence of others is necessary to define SA, we argue that, this in itself, is not sufficient. Otherwise, activities intuitively recognized as *not socially demanding*, may be mis-categorized as such. For example, a lecture is usually given to a group of attendees, rather than a single individual. While being a group-orientated activity, ‘attending a lecture’ is almost exclusively defined as a ‘cognitive’ rather than ‘social’ activity (Schultz et al., 2015; Seider et al., 2016; Vaughan et al., 2014; Vemuri et al., 2016, 2012). This could be attributed to it missing a potentially crucial component of SA, i.e. the *direct* interactions between individuals. More generally, these direct interactions could take the form of engaging in a conversation, providing emotional support and shared reminiscing, among other examples. Based on our discussion, we put forward the following criteria for SA: the (1) presence of others and (2) explicit communication between members participating in the activity.

**Fig. 6:**
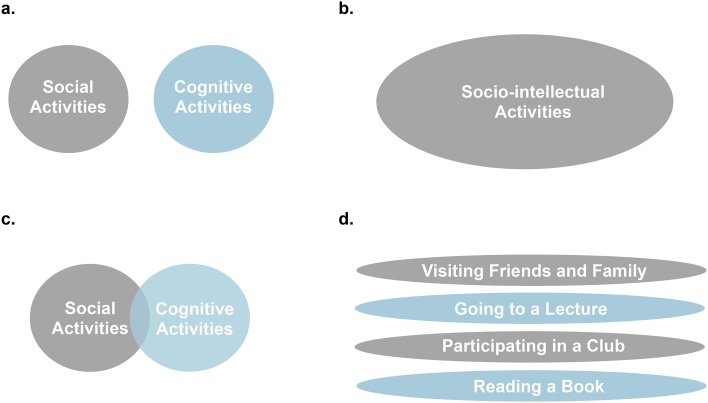
Schematic diagrams representing the different methods used to operationalize social (SA) and cognitive (CA) activities. **a.** SA and CA are treated as distinct constructs. **b.** SA and CA are combined into a single measure to reflect ‘socio-intellectual activities’. **c.** SA and CA are employed as distinct, but related constructs. **d.** Each activity item is examined as an independent correlate of the outcomes of interest.

The second methodological issue is the operationalization of socio-intellectual activities. Specifically, the reviewed evidence mainly utilized two approaches. The first involves using the distinct categories of CA and SA, where each consists of an independent cluster of activities (Fig. 6a). This method was employed by four of the included studies (see Table 1) and has the benefit of examining the individual effects of CA and SA. A major disadvantage is that it provides a poor fit for activities with both strong mental and interpersonal components (e.g. playing bridge; Cheng, 2016). Additionally, there was substantial disagreement among the reviewed studies on which particular activities fell under CA or SA. We sought to reduce these discrepancies by re-categorizing the reviewed studies in accordance with the distinctions of CA and SA suggested by Seider et al. (2016). One alternative to distinguishing between CA and SA is to examine ‘socio-intellectual activities’ as a singular construct (Fig. 6b), which was the method used by thirteen of the reviewed studies. This latter approach is advantageous over the former as it allows for activities to have concurrent social and cognitive demands, reduces inter-study disagreement and examines the combined effects of CA and SA. It is, however, not without its own set of problems. For example, it involves summing across a diverse range of activities, which has the risk of attenuating effects of interest and in extreme cases, producing null findings (Cheng, 2016). Relatedly, it prevents us from dis-entangling the potentially independent effects of socially and cognitively demanding activities on the aging brain. There are two additional methods that are currently underused by studies within this area, which may help to overcome the problems faced by the dichotomous and composite operationalizations of CA and SA. We suggest them as either substitutes or complimentary to currently employed approaches.

One of the proposed methods is to operationalize CA and SA as two related, but distinct constructs (Fig. 6c). In practice, this could involve rating each activity according to their perceived social and cognitive demands (e.g. low/ medium/ high), before combining activities with medium-to-high demands into CA and SA constructs. A similar approach was used in one of the reviewed studies (Köhncke et al., 2016) and has also been used in studies examining cognition (Singh-Manoux et al., 2003). This method addresses the problem presented by activities with concurrent cognitive and social demands, as such items could feature in both the CA and SA constructs. It also shares a key advantage of the dichotomous method, in that it can be used to investigate the independent effects of CA and SA. As with all composite measures, however, it still retains the risk of attenuating effects of interests. To address this issue, we suggest that a complimentary approach is applied, if the statistical power of a study allows for it. Specifically, each activity within the CA and SA constructs could be evaluated as independent correlates of the neuroimaging outcomes examined (Fig. 6d). Used by two of the included studies (Bennett et al., 2006; Schultz et al., 2015), this method may be particularly informative for intervention development. This is because it has the potential to highlight an activity (or set of activities) especially beneficial for brain health, and therefore identify potential targets for novel interventions. Overall, we propose that future studies should consider using the abovementioned approaches in their analyses, as they may aid the field in continuing to make sufficient progress towards developing interventions that promote healthy brain aging.

## 5 Conclusions

In the past decade, a growing number of MRI studies have started to focus on the relationship between CA and SA levels and the aging brain. Among the reviewed studies, we found that composite measures of CA and SA were related to global WM (volume and lesions) and hippocampal volume. Moreover, socio-intellectual activity levels correlated positively with regional GM volumes located within all major lobes of the brain. No link was found with global GM volume and studies examining WM microstructure produced inconsistent results. These findings are taken to suggest that leisure activities may contribute to *brain reserve*, with other neuroimaging studies also implicating a role in *cognitive reserve* and the development of AD-related neuropathology. As this field remains in its relative infancy, we propose several methodological improvements and avenues for further work. These suggestions have included (1) the use of multi-modal imaging and longitudinal RCTs, in addition to (2) wearable technology for the objective measurement of socio-intellectual activities. We also contribute (1) criteria for defining CA and SA and (2) suggest alternative methods to operationalize these constructs, which could be used by future studies. Overall, the current evidence supports a protective role of CA and SA against the negative effects of aging on GM and WM structures in the brain during late-life.

## Conflicts of interest

The authors declare no competing financial interests.
