# Supplementary material for: A systematic review and meta-analysis of structural magnetic resonance imaging studies investigating cognitive and social activity levels in older adults
Source: Neurosci Biobehav Rev. 2018 Oct;93:71–84. doi: 10.1016/j.neubiorev.2018.06.012 (PMC6562200; doi:10.1016/j.neubiorev.2018.06.012)
Supplement: Supplementary file 1 [file mmc1.docx]

**SUPPLEMENTARY MATERIAL**1. *Social Behavior/
2. Cognitive Activity.mp.

3. exp Leisure Activities/

4. lifestyle factors.mp.

5. 1 or 2 or 3 or 4

6. exp Magnetic Resonance Imaging/

7. exp Brain/

8. 5 and 6 and 7

9. limit 8 to (English language and humans, “all aged (65 and over)” and journal article)

**Figure S1.** Search criteria for MEDLINE.

**Figure S2.** Study selection process.

1 article identified through manual search

Studies included in meta-analysis:

Global Grey matter = 4

Global White matter = 4

Global White matter lesions = 5

Hippocampus = 7

489 articles identified through Medline, PsychINFO and EMBASE (28th December 2017)

432 articles excluded after abstract screening for the following reasons:
Not relevant to research question,
Ineligible sample

Social activities or Cognitive activities not measured
No MRI assessment

23 full-text articles assessed for eligibility

5 full-text articles excluded:

Ineligible sample

Unpublished

Associations between Social and Cognitive engagement and MRI measures not directly assessed

**Screening**

**Identification**

**Included**

**Eligibility**

18 studies included in systematic review

455 articles retained after duplicates removed

| **Cognitive Activities** | **Social Activities** |
| --- | --- |
| - Using a computer - Doing arts and crafts - Going to the cinema, concert, lecture or sport event - Playing a musical instrument - Reading | - Visiting family or friends - Visiting a senior centre - Volunteering - Attending church - Participating in groups or clubs - Playing cards or board games with others - Shooting pool or billiards |

**Table S1.** Cognitive and social activity distinctions suggested by Seider et al. (2016).


**Figure S3.** Cognitive and social activity engagement and global grey matter volume: a funnel plot of standard errors plotted against Fisher’s Z.


**Figure S4.** Cognitive and social activity engagement and hippocampal volume: a funnel plot of standard errors plotted against Fisher’s Z.

**Figure S5.** Cognitive and social activity engagement and global white matter volume: a funnel plot of standard errors plotted against Fisher’s Z.


**Figure S6.** Cognitive and social activity engagement and global white matter lesions: a funnel plot of standard errors plotted against Fisher’s Z.
